# Effect of Yerba Mate and Silk Fibroin Nanoparticles on the Migration Properties in Ethanolic Food Simulants and Composting Disintegrability of Recycled PLA Nanocomposites

**DOI:** 10.3390/polym13121925

**Published:** 2021-06-10

**Authors:** Freddys R. Beltrán, Marina P. Arrieta, Diego Elena Antón, Antonio A. Lozano-Pérez, José L. Cenis, Gerald Gaspar, María U. de la Orden, Joaquín Martínez Urreaga

**Affiliations:** 1 Departamento de Ingeniería Química Industrial y Medio Ambiente, Universidad Politécnica de Madrid, E.T.S.I. Industriales, 28006 Madrid, Spain; f.beltran@upm.es (F.R.B.); d.elena@alumnos.upm.es (D.E.A.); geraldmanuel.gaspar@upm.es (G.G.); joaquin.martinez@upm.es (J.M.U.); 2 Grupo de Investigación Polímeros Caracterización y Aplicaciones (POLCA), Madrid, Spain; mariula@ucm.es; 3 Depertamento de Biotecnología, Genómica y Mejora Vegetal, Instituto Murciano de Investigación y Desarrollo Agrario y Alimentario (IMIDA), 30150 Murcia, Spain; abel@um.es (A.A.L.-P.); josel.cenis@carm.es (J.L.C.); 4 Deparamento de Química Orgánica, Facultad de Óptica y Optometría, Universidad Complutense de Madrid, 28037 Madrid, Spain

**Keywords:** poly(lactic acid), nanocomposites, mechanical recycling, silk fibroin, yerba mate, migration, composting

## Abstract

The main objective of the present research is to study the effect of the incorporation of low amounts of silk fibroin nanoparticles (SFNs) and yerba mate nanoparticles (YMNs) on the migration phenomenon into ethanolic food simulants as well as on the disintegrability under composting conditions of mechanically recycled polylactic acid (PLA). Recycled PLA was obtained under simulated recycling conditions by melt processing virgin PLA into films and further subjecting them to an accelerated aging process, which involved photochemical, thermal, and hydrothermal aging steps followed by an intense washing step. SFNs were extracted from *Bombyx mori* cocoons and YMNs from yerba mate waste. Then, recycled PLA was melted, reprocessed, and reinforced with either 1%wt. of SFNs or YMNs, by melt extrusion, and further processed into films by compression molding. The obtained nanocomposites were exposed to ethanolic food simulants (ethanol 10% *v*/*v*, simulant A and ethanol 50% *v*/*v*, simulant D1) and the structural, thermal, and mechanical properties were studied before and after the exposure to the food simulants. The migration levels in both food simulants were below the overall migration limits required for food contact materials. The materials were disintegrated under simulated composting conditions at the laboratory scale level and it was observed that the nanoparticles delayed the disintegration rate of the recycled PLA matrix, but nanocomposites were fully disintegrated in less than one month.

## 1. Introduction

Poly(lactic acid) (PLA) is one of the most established bioplastics in the market due to its applications in industries such as the biomedical, automotive, and 3D printing industries and especially in the food packaging field [1,2]. PLA is an aliphatic polyester that has been proposed as an alternative with a lower environmental impact to fossil-fuel-based and non-biodegradable plastics, since it is produced from renewable resources such as corn, potato, and sugar beet [3,4] and is compostable [5,6]. Despite the environmental advantages of PLA, its growing production (680 kT forecasted for 2024 [7]) and high consumption in short-term applications [5,6] might cause some problems, mainly related to the management of the wastes generated after its use.

PLA is a biodegradable material; however, newer grades are very resistant and only biodegradable under industrial composting conditions, i.e., approximately 58 °C, a relative humidity of 60%, a pH ≅ 7.5, a C/N relationship between 20:1 and 40:1, and proper aeration [8,9]. Such conditions are not available under environmental conditions such as in landfills. Thus, inadequate management of PLA wastes could lead to their accumulation in the environment, with the negative impact it entails [10,11]. Furthermore, this linear use and disposal of valuable materials strongly disagrees with the circular economy approach proposed by the European Commission, in which resources are recirculated and used in a more sustainable way [12,13]. Therefore, it is necessary to develop, and implement, valorization methods that allow us to introduce PLA and other bioplastics into the circular economy approach. In this regard, mechanical recycling could play a very important role, since it allows us to reduce the consumption of raw materials, energy, and emissions associated with the production of PLA [14,15] and it also allows us to reduce the amount of PLA wastes in landfills.

Although mechanical recycling comes along with several advantages from an environmental point of view, it also poses several challenges, from both logistic and technical points of view. Nowadays, bioplastics are considered as contaminants in plastics recycling streams, since they cause a decrease in the performance of other plastics [16,17]. Therefore, it is necessary to adapt the current collection and sorting system to introduce a PLA stream. From a technical point view, previous studies have reported that mechanical recycling causes a decrease in the performance of PLA-based materials due to the degradation of the polymer [18], which negatively affects the recyclability of the material. In this regard, several methods have been proposed to improve the properties of mechanically recycled PLA, such as the use of chain extenders and crosslinking agents [19,20], the application of thermal treatments [21], and addition of inorganic [22] and organic fillers [23,24]. In the food packaging field, particular attention has been paid to PLA-based nanocomposites, since nanofillers lead to an improvement in the PLA’s thermomechanical and barrier performance, which are very important for packaging processing and manufacturing, as well as for the intended use [6,25].

It is worth noting that the addition of organic fillers coming from renewable resources could be an interesting method for the improvement of the properties of PLA due to the renewable origin, availability, low cost, and biodegradability of such fillers. Nanoparticles extracted from renewable resources (e.g., crops, arthropods) as well as those coming from agro-food wastes are of particular interest for sustainable materials within the frame of a circular economy approach. In this context, PLA has been widely blended with lignocellulosic nanoparticles as well as protein-based nanoparticles. For instance, the incorporation of lignocellulosic nanoparticles extracted from waste of yerba mate (*Ilex paraguariensis*), a commonly consumed infusion in South America, into a PLA matrix has proven to be an effective way to increase the crystallization rate of PLA, leading to an improvement in the mechanical performance of the final nanocomposites [26]. Yerba mate is rich in methylxanthines (caffeine and theobromine), but the well-known properties of yerba mate are attributed to its rich composition in bioactive substances such as phenolic compounds (e.g., lignin, caffeic acid, and chlorogenic acid), flavonoids (e.g., catechin, quercetin, rutin, and kaempferol), saponins, tannins, and some vitamins (e.g., C, B1, and B2) [27,28]. The use of yerba mate waste for the extraction of lignocellulosic nanoreinforcements with antioxidant activity has been shown to be an effective way to revalorize such waste and improve the thermal stability of PLA [26]. Interestingly, silk fibroin (SF), a natural biopolymer, also showed the ability to promote the crystallization of PLA-based materials, improving the mechanical and barrier performance of the final nanocomposites [29]. Silk fibroin is a fibrous protein obtained from *Bombyx mori* cocoons and has been used by the textile industry for more than 8500 years [30] due to its excellent physical properties, such as light weight, high mechanical strength, flexibility, and luster. Over the last few decades, a wide range of new applications have been developed based on its biocompatibility, biodegradability, and non-immunogenicity, becoming an outstanding candidate for biomedical uses [31]. Additionally, in previous works the addition of small amounts (e.g., 1 wt.%) of lignocellulosic nanoparticles extracted from yerba mate [23] as well as silk fibroin nanoparticles [24] produced an improvement in the overall properties of mechanically recycled PLA mainly because a good dispersion of the nanoparticles into the polymeric matrix was achieved. Meanwhile, the incorporation of 3 wt.% of yerba mate nanoparticles tend to aggregate [23]. However, the control of the release of potentially migrant compounds from the packaging material to the foodstuff is of fundamental importance in PLA-based food packaging materials. In previous works, it was observed that the low-molecular-weight compounds, such as plasticizers, favor the release of components from the PLA-based packaging materials to the foodstuff [32,33]. Thus, in the case of mechanically recycled PLA materials, in which the polymer undergoes degradation during the service and recycling process leading to a reduction in the molecular weight with the formation of shorter polymeric chains, an increase in the migration phenomenon is expected. Nevertheless, different results were obtained for recycled PLA-based nanocomposites reinforced with yerba mate and silk fibroin nanoparticles in which the homogeneous distribution of nanoparticles into the polymeric matrix leads to materials with improved overall performance [23,24], and thus with potentially more complex migration processes.

Consequently, the main objective of the present research is to study the effect of the addition of low amounts (1 wt.%) of either YMNs or SFNs on the migration phenomenon of recycled PLA exposed to two ethanolic food simulants: a hydrophilic food simulant (food simulant A: 10% *v*/*v* ethanol solution) and a fatty food simulant (simulant D1: 50% *v*/*v* ethanol solution) as well as the effect of such nanoparticles on the bionanocomposites’ disintegration under composting conditions. This would allow us to obtain information regarding the use of these nanocomposites in the food packaging field as well as provide a sustainable end-of-life option to these recycled PLA-based nanocomposites after their useful life.

## 2. Materials and Methods

### 2.1. Materials

A commercial grade of PLA designed for packaging applications, the Ingeo^TM^ 2003D, was purchased from NatureWorks (Minnetonka, MN, USA). This grade presents a specific viscosity of 1.24 and a melt-flow rate of 6 g/10 min (210 °C, 2.16 kg). The yerba mate (*Ilex paraguariensis*) residues were collected after consumption of a yerba mate infusion in our laboratory. *Bombyx mori* cocoons were supplied by Instituto Murciano de Investigación y Desarrollo Agrario y Alimentario (IMIDA, Murcia, Spain). All chemicals and solvents were purchased from Merck (Madrid, Spain), were of analytical grade, and were used without further purification, unless otherwise specified.

### 2.2. Extraction of Yerba Mate Nanoparticles (YMN)

**Figure 1:**
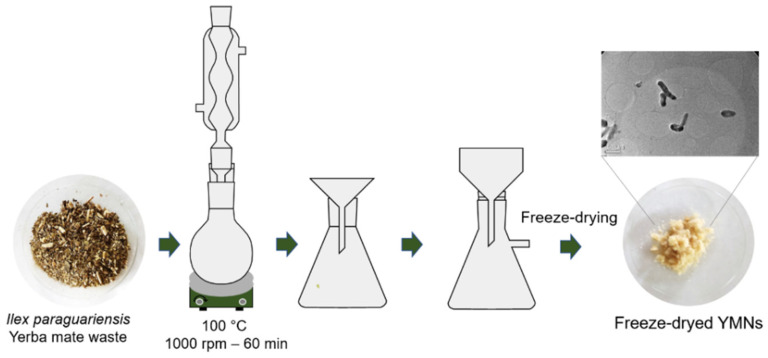
Schematic representation of the yerba mate nanoparticles (YMN)’ extraction from yerba mate infusion wastes.

The extraction of the yerba mate nanoparticles from yerba mate waste was conducted according to the procedure developed by Arrieta et al. [26], which is shown in Figure 1: (i) the yerba mate waste was firstly dried in an oven (24 h at 60 °C); (ii) 100 mL of distilled water was added to 6 g of the dry yerba mate residue and heated under reflux up to 100 °C and under continuous stirring; (iii) the solid residue was separated by filtration, and the extracted yerba mate solution was filtered again using Whatman Grade 41 filter paper; and (iv) the filtered solution was then frozen and freeze-dried to obtain the YMN powder. Dynamic light scattering (DLS) measurements showed that the YMNs in solution present an average size of 93 nm (Figure S1a) and the YMNs in powder present an average size of 497 nm (Figure S1a) due to the fact that YMNs tend to agglomerate during the freeze-drying process [23] as can be seen in the TEM image (Figure S1b).

### 2.3. Obtention of the Silk Fibroin Nanoparticles (SFN)

**Figure 2:**
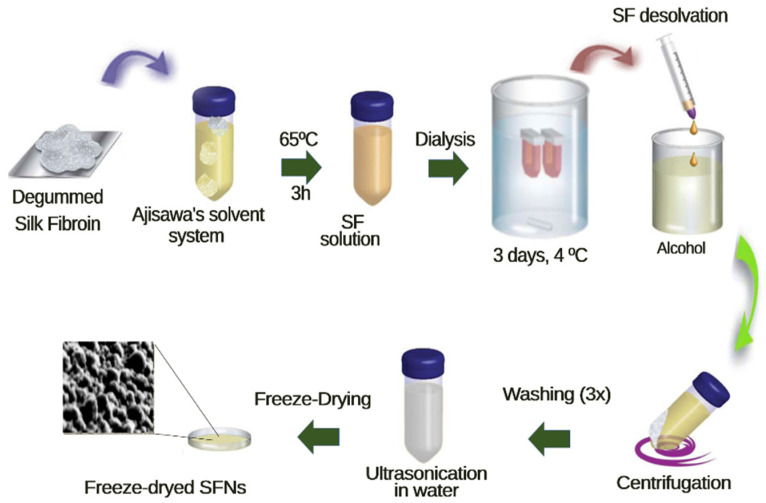
Schematic representation of the silk fibroin nanoparticles (SFN) preparation.

The silk fibroin nanoparticles were prepared by rapid desolvation of the freshly prepared silk fibroin aqueous solution in absolute ethanol, following the procedure described previously by Lozano-Pérez et al. [34]. In brief, degummed silk fibroin [35] was dissolved in Ajisawa’s solvent system at 65 °C for 3 h [36], filtered, and dialyzed for 48 h against distilled water with several changes in order to remove salts and alcohol. Then, the aqueous silk fibroin solution was dripped on ethanol under gentle stirring, resulting in the formation of the nanoparticles by rapid desolvation. The resultant suspension was stirred for 2 h, until the nanoparticles were stabilized, and then were recovered by centrifugation at 12,000× *g* for 15 min and 8 °C in an Eppendorf 5810R centrifuge (Eppendorf AG, Hamburg, Germany) equipped with an F-34-6-38 rotor, repeatedly washed (3×) with ultrapure water (18 MΩ·cm, from a Purelab Flex 2 ultrapure water Type I system, ELGA, High Wycombe, UK), and finally dispersed in ultrapure water by using high-power ultrasounds for 1 min at 10% of amplitude in a Branson SFX 550 Digital Sonifier equipped with a 1/8″ tapered microtip (Branson Ultrasonics Corp, Ridge Road Brookfield, CT, USA) (Figure S2). The resulting dispersion was then freeze-dried at −55 °C and 0.05 mbar for 72 h in a Christ Alpha 1-2 LDplus freeze-dryer (Martin Christ Gefriertrocknungsanlagen GmbH, Osterode am Harz, Germany) to obtain the SFNs as a white powder. A scheme of the process can be seen in Figure 2.

### 2.4. Mechanical Recycling of PLA and Preparation of the Nanocomposites

**Figure 3:**
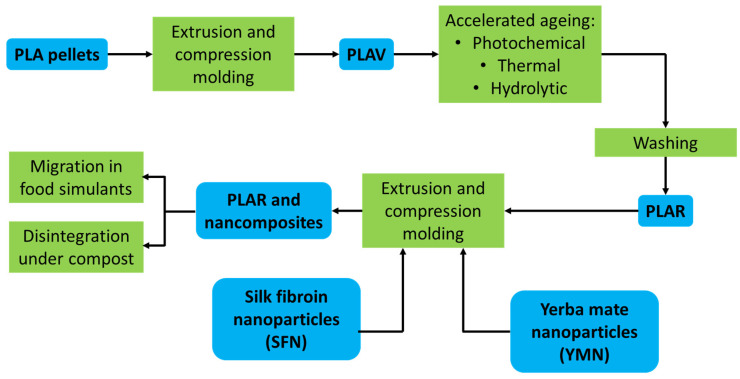
Scheme of the recycling of poly(lactic acid) (PLA) and the manufacturing of nanocomposites.

Figure 3 shows the schematized mechanical recycling process along with the preparation of the nanocomposites. The number average molecular weight (Mn) and polydispersity indexes (PDI) of PLA pellets as well as PLA and recycled PLA film were measured by Gel permeation chromatography (GPC) using a Waters Division Millipore chromatographic system (Milford, MA, USA) equipped with a Waters 2414 refractive index detector and Styragel columns and using THF (HPLC grade, Fisher Scientific, Madrid, Spain) (1 mL min^−1^ flow rate) as an eluent at 40 °C. The calibration was made with polystyrene standards.

In a first stage, PLA pellets were thoroughly dried (2 h at 85 °C and under vacuum) (Mn = 109,000 and PDI = 1.6) and melt-processed in a Rondol Microlab twin screw micro-compounder (Nancy, France) (L/D ratio = 20), at 60 rpm, with the following temperature profile from hopper to die: 125–165–190–190–180 °C. The resulting material was then transformed into films (thickness = 200 ± 10 µm) using a hot plate press (IQAP-LAP, Barcelona, Spain) at 190 °C for 5 min. In a second stage, the obtained films were subjected to an accelerated aging protocol, including 40 h of photochemical aging in an UV-CON chamber (Atlas, Mount Prospect, IL, USA) equipped with 8 F40UVB lamps, 280 h of thermal aging in an oven at 50 °C, and 240 h of hydrothermal aging at 25 °C in demineralized water. Then, the aged samples were washed at 85 °C in a solution of NaOH (1.0 wt.%) and Triton X-100 (Alfa Aesar, Kandel, Germany) (0.3%wt.) following the conditions used in previously published recycling studies [18,37]. In a third stage, the washed material was shredded and processed into recycled PLA film (PLAR, Mn = 80,500 and PDI = 1.8), blended with YMNs (1 wt.%) and SFNs (1 wt.%), and melt-extruded to be further compression molded under the same conditions as PLAV (PLAV, Mn = 92,000 and PDI = 1.8). The obtained nanocomposites showed a homogeneous distribution of the nanoparticles into the polymeric matrix (Figure S3). The obtained materials were finally subjected to migration studies in food simulants and to disintegration under composting conditions tests. Table 1 summarizes the obtained samples.

### 2.5. Overall Migration Tests

Overall migration tests of the different samples were performed in two different ethanolic food simulants [38]: simulant A, a 10% *v*/*v* ethanol solution; and simulant D1, a 50% *v*/*v* ethanol solution (ethanol absolute, Panreac, Barcelona, Spain). The samples areas exposed to the food simulants were selected on the basis of the overall migration test standard [39]. Four specimens of 1.5 × 1.5 cm were immersed in 9 mL of food simulant (area-to-volume ratio = 1 dm^2^/100 mL) contained in glass vials. The immersed samples were kept in an oven at 40 °C for 10 days. Then, the immersed samples were removed and thoroughly dried for characterization. Meanwhile, the glass vials were heated on a heating pan to evaporate most of the food simulant and then dried in a vacuum oven at 70 °C for 1 day. The dry vials were weighted in a QUINTIX125D-1S analytical balance (Sartorius, Gotinga, Germany) to determine the amount of migrated material.

### 2.6. Disintegration under Composting Conditions

The different samples were disintegrated under simulated composting conditions at laboratory scale, according to the ISO 20,200 standard [40]. Specimens of 1.5 × 1.5 cm were buried at 4-6 cm deep in a perforated plastic container containing solid synthetic waste. Samples were kept in textile mesh to allow for the removal of disintegrated film samples during different days of the composting test as well as to be in direct contact with the compost medium [41]. The solid synthetic waste was formed by mature compost (Compo Group, Barcelona, Spain) (10 wt.%), sawdust (Productos Adrian, Valencia, Spain) (40 wt.%), rabbit food (Vitakraft, Bremen, Germany) (30 wt.%), starch (Unilever Spain, Barcelona, Spain) (10 wt.%), sugar (Azucarera, Madrid, Spain) (5 wt.%), corn oil (Koipe, Córdoba, Spain) (4 wt.%), and urea (Quimipur, Madrid, Spain) (1 wt.%). Up to 50 wt.% water was then added to the synthetic waste. The containers were kept under aerobic conditions at 58 °C in an oven (Memmert GmbH, Schwabach, Germany). Specimens were removed at 1, 4, 11, and 17 days. The degree of disintegrability was calculated following Equation (1):

(1) $$\%\;\mathrm{disintegration}=\frac{w_{0}-w_{t}}{w_{0}}\times 100$$

where *w*_0_ is the initial weight of the specimens and *w_t_* the weight after *t* days buried in the composting medium.

The recovered specimens were then dried and stored under vacuum for characterization.

### 2.7. Characterization Techniques

Differential scanning calorimetry tests were conducted on a Q20 calorimeter (TA Instruments, New Castle, DE, USA) under a nitrogen atmosphere. Samples (5 mg) were put in standard aluminum pans and subjected to a first heating scan, at 5 °C/min, from 30 to 180 °C; an isothermal step at 180 °C for 3 min; a cooling scan, at 5 °C/min, from 180 to 0 °C; and an isothermal step at 0 °C for 1 min, and a second heating scan, at 5 °C/min, from 0 to 180 °C. The crystallinity degree, $X_{C}^{DSC}$, was calculated using Equation (2):

(2) $$X_{C}^{DSC}=\frac{\Delta H_{m}-\Delta H_{cc}}{\Delta H_{\infty }}\times 100$$

where ∆*H_m_* and ∆*H_cc_* are the melting and cold crystallization enthalpies, respectively.

Fourier Transform Infrared (FTIR) spectra of the materials were recorded in a Nicolet iS10 spectrometer (Thermo Scientific, Waltham, MA, USA) equipped with a Smart iTR Attenuated Total Reflectance (ATR) accessory controlled by OMNIC Software Ver. 9.9.473. The resolution used was 4 cm^−1^, and 16 spectra were recorded for each sample. All the spectra were normalized at 1451 cm^−1^, a commonly used internal standard. The surface crystallinity degree was calculated using the 956 cm^−1^ absorption band according to Equation (3) [42]:

(3) $$X_{C}^{FTIR}=\frac{I_{0}-I_{t}}{I_{0}}\times 100$$

where *I*_0_ and *I_t_* are the intensity of the 956 cm^−1^ absorption band before and after the immersion in the different food simulants, respectively.

Thermogravimetric analysis of the samples was performed in a TGA2050 thermobalance (TA Instruments, New Castle, DE, USA) under a nitrogen atmosphere. Samples (10 mg) were put in a platinum crucible and heated from 40 to 800 °C at 10 °C/min.

Vickers hardness was measured in a Shimadzu Type M microhardness tester (Kioto, Japan) with a load of 25 g and a loading time of 10 s. Each sample was measured six times.

Other techniques used for nanoparticle characterization are detailed in the supplementary information file.

### 2.8. Statistical Analysis

Significance in overall migration studies as well as in mechanical properties was analyzed using one-way analysis of variance (ANOVA) with Origin Pro 8 software SRO v8.0724 (OriginLab Corporation, Northampton, MA, USA). Comparisons between groups were made by performing a Tuckey’s test with a 95% confidence interval. Thus, the reported *p*-values were considered statistically significant at *p* < 0.05.

## 3. Results and Discussion

### 3.1. Migration to the Food Simulants

**Figure 4:**
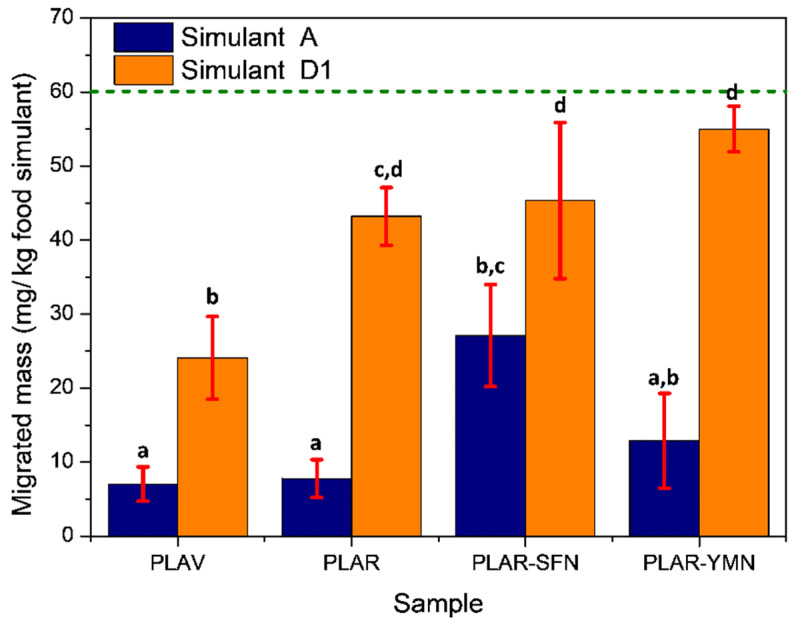
Overall migration of virgin poly(lactic acid) (PLAV) and recycled poly(lactic acid) (PLAR), and nanocomposites to the different food simulants A) (ethanol 10% *v*/*v*) and D1) (ethanol 50% *v*/*v*). The green line indicates the legal overall migration limit (60 mg/kg of food simulant). ^a–d^ Different letters indicate statistically significant differences between formulations (*p* < 0.05).

Overall migration studies were conducted in food simulants A and D1 and the results after 10 contact days are shown in Figure 4. Higher migrations levels were observed in simulant D1 with respect to the same material exposed to simulant A (*p* < 0.05). With increasing the ethanol concentration from 10% *v*/*v* in Simulant A to 50% *v*/*v* in Simulant D1, higher migration values were observed in all formulations (*p* < 0.05). It is known that sorption of certain organic solvents, including ethanol, causes swelling of the PLA matrix and creates voids enlarging the gaps between molecules in the polymer structure, resulting in an increase in the free volume that further promotes the diffusion of potential migrants (additives, low-molecular-weight compounds, etc.) from the polymer to the food simulant [43,44,45]. Higher migration levels were observed in the PLAR sample with respect to PLAV in Simulant D1 (*p* < 0.05), in which the highest amount of ethanol promotes a higher free volume increment. This could be explained by the degradation of PLA during recycling, which generates shorter polymer chains. Migration of these low-molecular-weight segments could be more easily promoted by ethanol, while more water molecules are able to diffuse into the polymeric matrix, accelerating the hydrolysis process. With regard to the nanocomposites, somewhat higher migration levels (*p* > 0.05) were observed, but it should be highlighted that all the materials presented migration levels lower than the legal overall migration limit of 60 mg/kg of food simulant [38].

### 3.2. Structural Changes after Immersion in the Food Simulants

**Figure 5:**
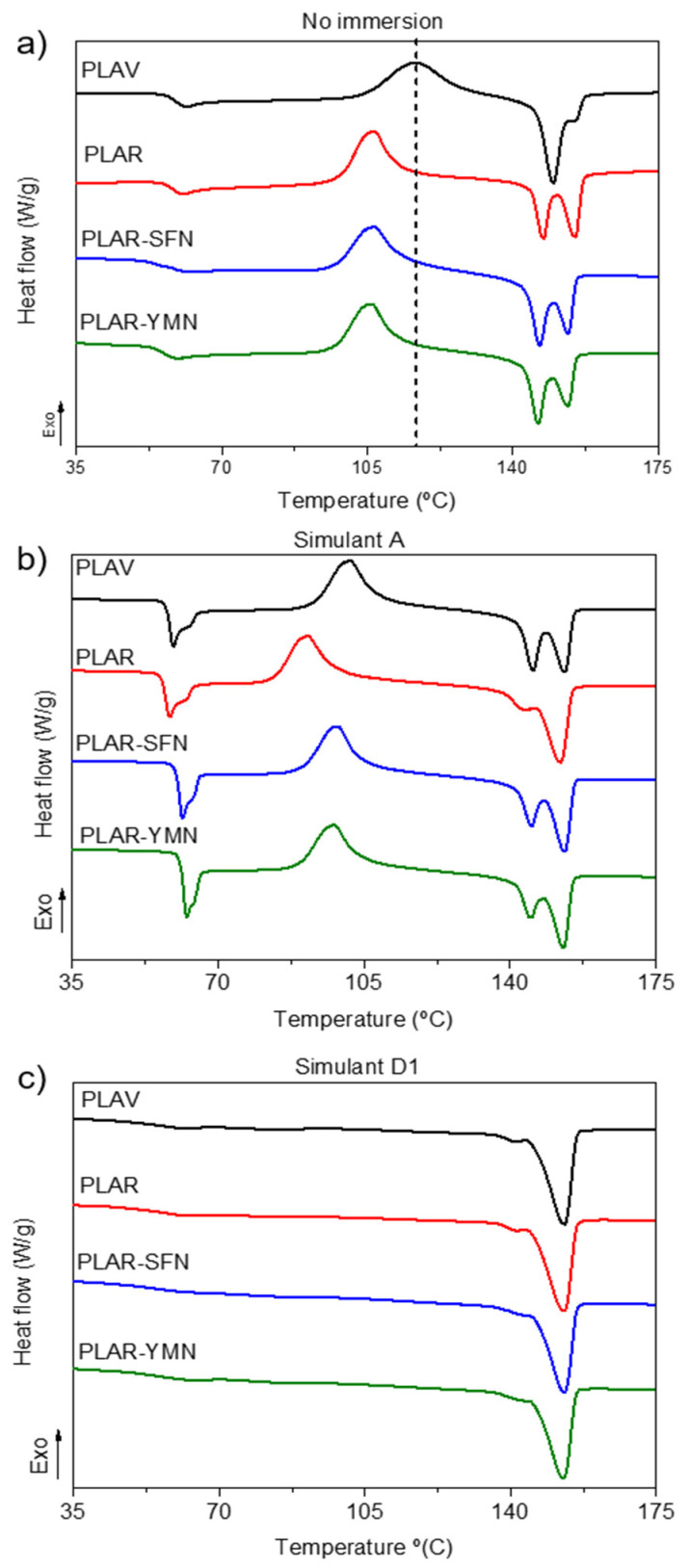
DSC first heating scans of the samples before (**a**) and after overall migration tests in food simulants: A (ethanol 10% *v*/*v*) (**b**) and D1 (ethanol 50% *v*/*v*) (**c**).

The degradation and subsequent migration of the degradation products to the food simulant are not the only effects of ethanolic solutions on PLA matrix. Previous studies, such as those conducted by Fortunati et al. [43], Arrieta et al. [32], and Iñiguez-Franco et al. [44,46], have reported an increase in the crystallinity degree of PLA-based materials after immersion in ethanolic solutions at different concentrations. The structural changes of the different samples after immersion were studied by means of DSC and FTIR spectroscopy. Figure 5 shows the DSC first heating scans before immersion (a) and after the immersion in simulant A (b) and simulant D1 (c). Table 2 summarizes the DSC results of the different samples.

Figure 5a shows that before immersion, all the samples showed the characteristic thermal transitions of PLA: (i) a glass transition between 55 and 60 °C; (ii) a cold crystallization exothermic peak above 100 °C; and (iii) a melting endotherm above 140 °C. Despite the similar overall behavior of all the materials, there are some important differences between the recycled PLA-based materials and PLAV. Both Figure 5a and Table 2 show that mechanical recycling caused an important decrease in the *T_cc_* value of PLA, which can be attributed to the presence of shorter polymer chains with higher mobility, thus forming crystalline structures at lower temperatures [18,47]. Regarding nanocomposites, it is expected that nanoparticles act as nucleating agents for PLAR, decreasing *T_cc_*, but this was not observed. This behavior could be ascribed to the nanoparticles hindering the movement of the molecular segments, thus countering the effect of the shorter polymer chains.

Moreover, noticeable differences can be observed in the melting endotherms of the different samples. The recycled PLA-based materials showed two well-defined melting peaks, attributed to the melting of two different crystal size populations, while PLAV showed a single peak accompanied by a high temperature shoulder. This difference could also be explained by the presence of shorter polymer chains in the recycled materials, since their higher mobility could allow them to rearrange into less-ordered crystalline structures during heating, and then melt at a higher temperature. This mechanism has been previously reported by Di Lorenzo et al. [48] in PLA-based materials. It is also worth noting that the addition of 1 wt.% of the different organic nanofillers only had a limited effect on the thermal transitions of recycled PLA, as has previously been commented and also reported in the literature [23,24].

Regarding the behavior of the samples after immersion in food simulant A, Figure 5b shows that, in all samples, an endothermic peak appeared in the glass transition region. This peak is ascribed to the physical aging of PLA, which consists in the slow rearrangement of polymer segments at temperatures below *T_g_*, resulting in a reduced free volume, higher stiffness, and decreased deformability [49,50]. In the conditions of this experiment, the increased chain mobility, due to the plasticizing effect of water, might have increased the rate of the physical aging process of all the samples, so the peak is more intense than in the starting materials [51,52]. Table 2 shows that, after the immersion in food simulant A, all the samples presented significantly lower *T_cc_* values than the non-immersed materials. This decrease in the cold crystallization temperature is related to the hydrolytic degradation of PLA, since shorter polymer chains can crystallize at lower temperatures. Figure 5b and Table 2 also show that PLAV has higher Tcc values than the PLAR-based materials due to the presence of larger polymer chains. However, the nanocomposites show higher *T_cc_* values than PLAR. This behavior could be due to the presence of the nanoparticles limiting the movement of molecular segments, thus hindering the formation of crystalline domains. A similar behavior was reported in PLA–halloysite nanocomposites immersed in a phosphate buffer solution at 58 °C [53].

It can also be seen in Table 2 that all the samples showed a slight crystallization, which could be attributed both to the plasticizing effect of the water and the hydrolytic degradation of the amorphous zones of PLA [44] as well as the above-described swelling effect produced by the ethanolic solution.

In the case of the samples immersed in the simulant D1, the structural changes are more drastic. Table 2 shows a slight reduction in the *T_g_* values, which could be due both to the severe degradation of PLA and the plasticizing effect of the ethanolic food simulants. Furthermore, it can be seen in Figure 5c that all the samples showed no cold crystallization peak. This behavior might indicate that all the available regions for crystallization were indeed crystallized during the immersion in the food simulant. This behavior is not uncommon in PLA-based materials immersed in aqueous solutions, as has been reported by authors such as Badía et al. [54] and Simmons et al. [55]. This significant crystallization could be related to the plasticizing effect produced by water and the swelling effect of the amorphous phase produced by ethanol, and also the severe degradation of the samples, leading to an induced crystallinity when exposed to 50% of each solvent as was pointed out by Iñiguez-Franco et al. [44,46].

**Figure 6:**
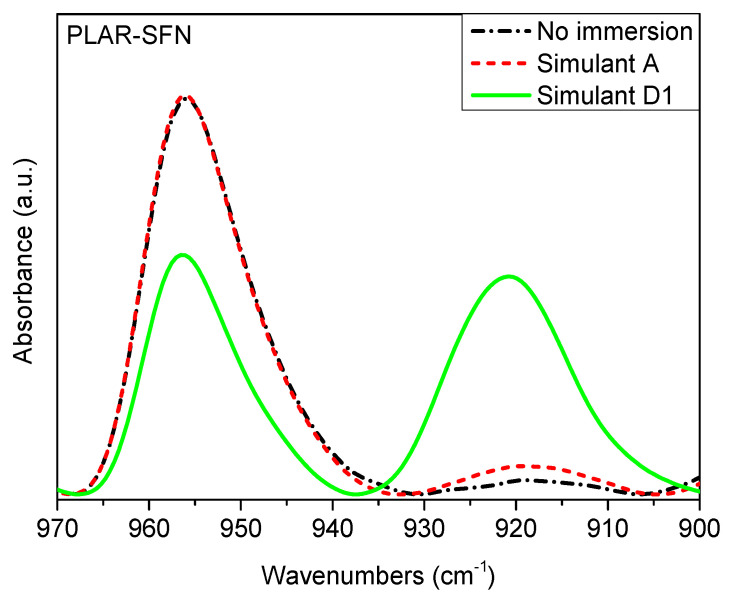
ATR-FTIR spectra of the recycled poly(lactic acid) and silk fibroin nanoparticle nanocomposite (PLAR–SFN) before and after immersion in the different food simulants. Region between 900 and 970 cm^−1^.

Additional information about the structural changes that took place on the surface of the samples, during the immersion in food simulants, can be obtained by means of ATR-FTIR spectroscopy. Figure 6 shows the region between 900 and 980 cm^−1^ of the ATR-FTIR spectra of PLAR before and after immersion in the different simulants. The absorption bands located at 956 and 920 cm^−1^ are assigned to the coupling of the stretching of the skeletal C-C bonds and the rocking of the CH_3_ [56]. The band located at 920 cm^−1^ can be correlated to the 10_3_ helix chain conformation characteristic of the ordered crystalline zones, while the band at 956 cm^−1^ can be assigned to the amorphous regions of the polymer [53,57]. As can be seen in Figure 6, the immersion in food simulants led to an increase in the absorption of the band centered at 920 cm^−1^, along with a decrease that centered at 956 cm^−1^, indicating a certain degree of crystallization, especially in the samples immersed in simulant D1. According to Meaurio el al. [42] and Beltran et al. [53], the degree of crystallinity can be estimated following the evolution of the band at 956 cm^−1^ Equation (3). Table 2 shows the values of crystallinity calculated with Equation (3), and it can be seen that no increase in the superficial crystallinity occurred during the immersion in food simulant A, with exception of the PLAR–SFNs nanocomposite, which showed a slight crystallization on its surface. On the other hand, Table 2 shows that all the samples immersed in food simulant D1 had a crystallization degree over 20%, in good agreement with the DSC results. Nevertheless, it can be seen that the nanocomposites showed lower surface crystallinity values than the unfilled PLA samples, especially the PLAR–YMNs sample. This result suggests that the presence of the nanoparticles inhibits the formation of crystalline domains on the surface of the nanocomposites while the global crystallinity, measured by DSC, is not affected by both nanoparticles.

### 3.3. Variation in the Properties of the Materials as a Result of the Immersion in Food Simulants

**Figure 7:**
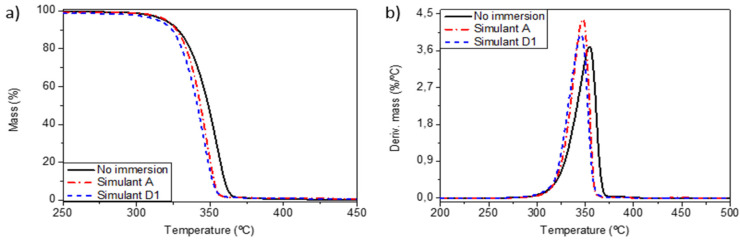
Weight loss (**a**) and derivative weight loss (**b**) of PLAV before and after immersion.

As has been discussed in the previous sections, the immersion of PLA in the different food simulants could cause important structural changes that can affect the properties of the materials. Thermal stability and hardness were measured, before and after immersion, by means of dynamic TGA and microhardness measurement tests, respectively. Figure 7 and Table 3 summarize the TGA results of the different samples.

Figure 7 shows that PLAV presented a single-step thermal degradation process. This behavior was followed by all the materials. The T_10_ values shown in Table 3 point out that mechanical recycling caused a slight decrease in the thermal stability of PLA as a result of the degradation of the polymer [18]. Nevertheless, the addition of SFNs and YMNs led to a slight increase in the thermal stability of the recycled material. This improvement in the thermal stability has already been observed for PLA reinforced with both YMNs [23,26] and SFNs [29]. The beneficious effect of the nanoparticles could be explained by their barrier effect, which limits the liberation of degradation products and increases the thermal stability of the polymeric matrix [24].

Table 3 also shows that, overall, the immersion in the different ethanolic food simulants resulted in a decrease in the thermal stability of all the samples, especially in the case of the simulant D1. These results could be explained by the hydrolytic degradation of the material during the immersion, since shorter polymer chains decompose at lower temperatures. These findings are in good agreement with those reported by Fukushima et al. [58] and Beltrán et al. [59] in PLA-based materials subjected to severe hydrolytic degradation conditions at 58 °C. Moreover, it is expected that the higher amount of ethanol in this food simulant extracts higher amounts of migrants from the packaging materials, leading to a higher reduction in the thermal stability. In fact, the reduction in the thermal stability was particularly marked in the YMN-based nanocomposite immersed in the food simulant D1. This behavior can be ascribed to a higher extraction of YMNs by the ethanol fraction followed by a solubilization effect of YMNs in the food simulant D1. In fact, ethanolic solutions are used to extract active components from yerba mate [60]

**Figure 8:**
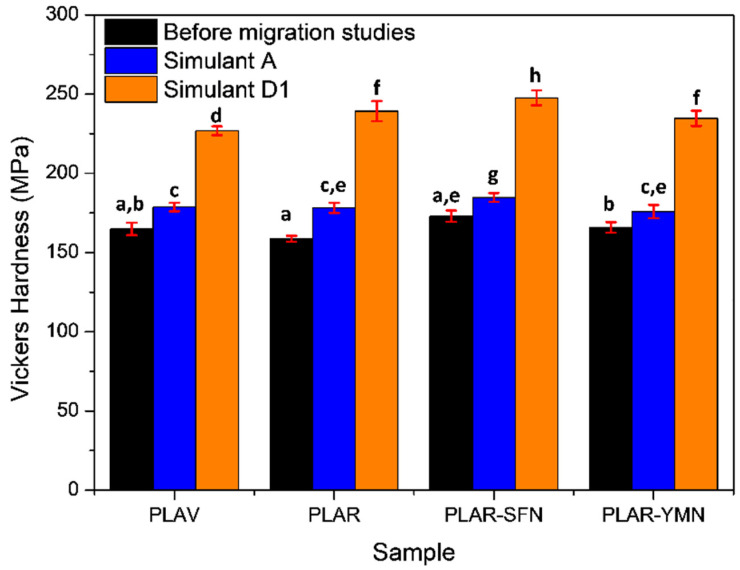
Vickers hardness of the samples before and after immersion in food simulants. ^a–h^ Different letters indicate statistically significant differences between formulations (*p* < 0.05).

The mechanical properties of the samples could also be affected by the structural changes that take place during the immersion in the different food simulants. Figure 8 shows the Vickers hardness values of the different samples before and after the immersion in the different food simulants. The mechanical recycling process led to a slight, but not significant (*p* > 0.05), decrease in the hardness of PLA. This behavior has been reported in previous studies, and it is related to the degradation of the polymer during the accelerated aging, washing, and melt reprocessing [18,61]. However, the addition of both silk fibroin and yerba mate nanoparticles led to a significant increase (*p* < 0.05) in the hardness of recycled PLA. The reinforcing character of these nanoparticles has already been pointed out in previous studies [23,24].

Regarding the behavior of the samples immersed in both food simulants, Figure 8 shows a significant increase in the Vickers hardness after the immersion for all the materials (*p* < 0.05). These results might seem surprising, since degradation reactions take place during immersion. In fact, an increase in the hardness of PLA after exposure to ethanolic food simulants has been already reported [32]. Indeed, hardness is not only affected by molecular weight, but also by other factors such as molecular configuration and crystallinity degree [62]. In the case of the sample immersed in simulant A, an increase of around 7% (*p* < 0.05) in the Vickers hardness was observed, although no important crystallization was determined by means of DSC or ATR-FTIR spectroscopy. This behavior suggests that physical aging, which was observed by means of DSC, is the main reason for the increase in the hardness of the immersed samples. Authors such as Wang and Mano [63], Pan et al. [64], and Cui et al. [49] have pointed out that physical aging, in dry conditions, of PLA leads to an increase in the hardness and stiffness of the polymer. Figure 8 also shows that the samples immersed in the simulant D1 presented a larger increase in the Vickers hardness (*p* < 0.05), which can be attributed to the important increase in the crystallinity degree upon immersion, as was observed by means of DSC and ATR-FTIR spectroscopy.

### 3.4. Disintegration under Industrial Composting Conditions

**Figure 9:**
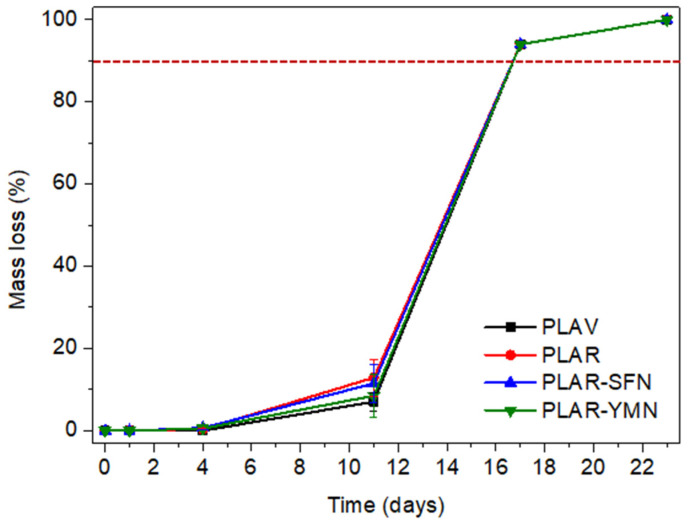
Degree of disintegration of films under composting conditions as a function of time.

**Figure 10:**
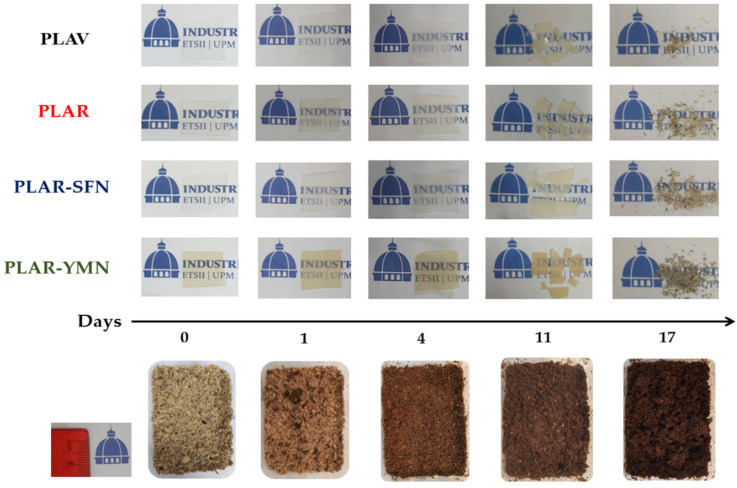
Visual appearance of the films and the compost medium during the composting test.

**Figure 11:**
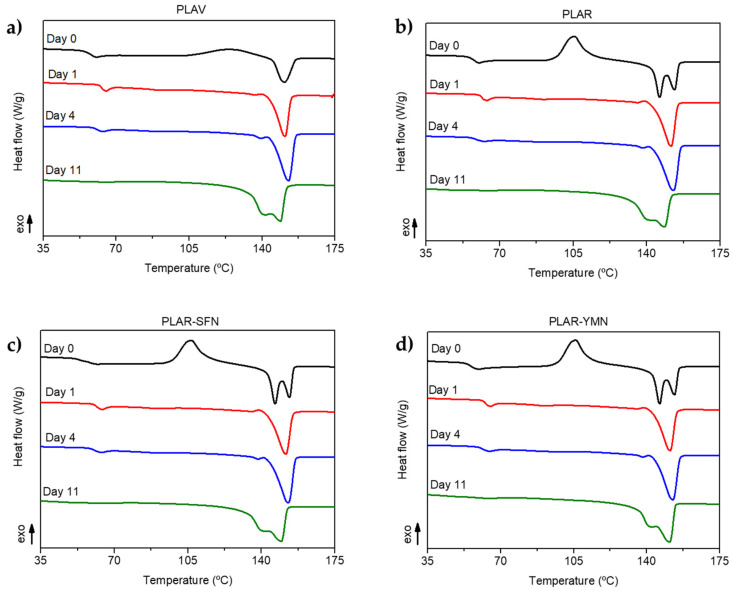
DSC first heating scan of (**a**) virgin PLA (PLAV), (**b**) recycled PLA (PLAR), (**c**) recycled PLA-silk fibroin nanoparticles nanocomposite PLAR-SFN, and (**d**) recycled PLA-yerba mate nanoparticles nanocomposite PLAR-YMN before and after different days of disintegration under composting conditions.

**Figure 12:**
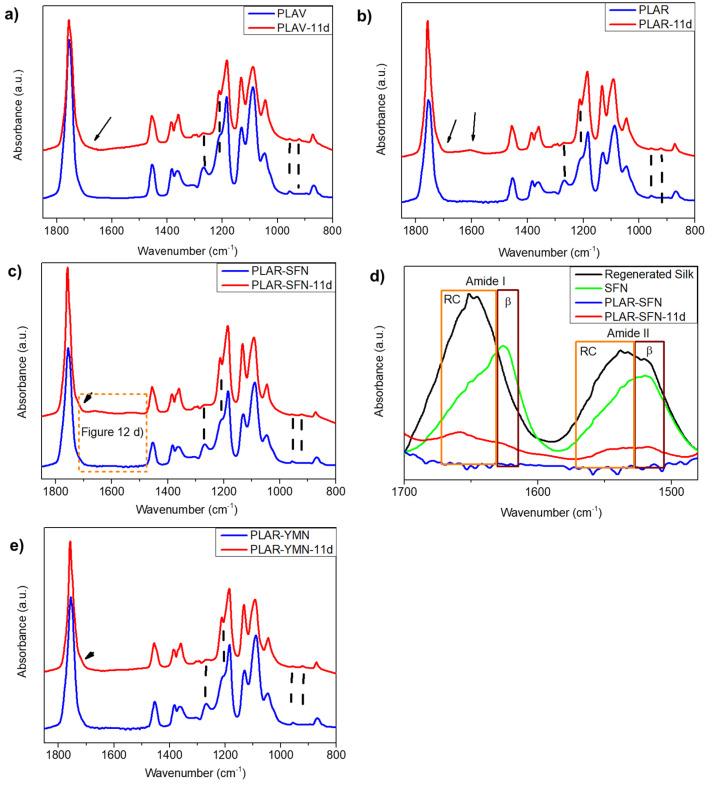
ATR-FTIR spectra of (**a**) PLA, (**b**) PLAR, (**c**) PLAR–SFNs, (**d**) the amide region of the PLAR–SFNs (spectra of regenerated silk, black, and SFNs, green, were added for reference), and (**e**) PLAR–YMNs before and after 11 days of disintegration under composting conditions.

As PLA cannot be infinitely recycled, due to each reprocessing cycle negatively affecting the thermomechanical properties of the PLA matrix [65,66], the recycled PLA-based materials after their useful life can be composted under industrial composting conditions in the same way as single-use PLA-based materials [67]. The degree of physical disintegration as a function of time was quantitatively monitored by the determination of samples’ mass loss, as is shown in Figure 9. Meanwhile, photographs were taken to qualitatively check the visual appearance of the recovered films at different composting times (Figure 10). A reduction in the molecular weight of the films during composting was followed by a reduction in the intrinsic viscosity (Table 4). The structural and chemical changes were followed by means of DSC (Figure 11) and ATR-FTIR (Figure 12).

Figure 10 shows that, after 4 days of composting disintegration, all the samples increased their opacity. However, no significant changes were observed in the mass loss among film formulations after 4 days of composting disintegration. The disintegrability of PLA under composting conditions starts by a hydrolysis process and it is further followed by enzymatic actions of microorganisms [8,41]. The increased opacity of PLA indicates that the hydrolytic disintegration process has started, since the water absorption and the presence of products generated during the hydrolytic degradation produce changes in the refraction index of polymeric materials [68]. Although no significant mass losses were observed, the PLAV and PLAR samples exhibited a reduction in the molecular weight after 4 days, as can be seen from the intrinsic viscosity reduction of about 42% (Table 4). A smaller reduction in the molecular weight was observed in the nanocomposites (around 33%). This result suggests that nanoparticles protect the polymeric matrix from the degradation process, due to the above-described barrier effect, which restricts the water penetration and delays the disintegration process. A similar behavior has already been observed in previous works, and it has been ascribed to the nucleating effect of such nanoparticles on PLA matrix [26]. In fact, TGA results show that the reduction in T_10_ values after 4 days in composting was around 45 °C in neat PLA, between 27 °C and 29 °C in PLAR and PLAR–SFNs, and around 15 °C for PLAR–YMNs. This lesser reduction in the T_10_ values may be related to the antioxidant effect of YMNs provided by the high amount of total polyphenol content (TPC of YMNs = 41 ± 1 mg of gallic acid/g yerba mate nanoparticles [26]), making YMNs able to protect the polymeric matrix from thermal degradation. The large decrease in the thermal stability after only 4 days of degradation was probably due to the loss of low-molecular-mass compounds, which were generated by hydrolysis during the initial degradation stage.

Figure 11 shows that after only one day in compost all the formulations crystallized, as the endotherm of the cold crystallization disappeared. After 11 days of composting, the glass transition peak disappeared, which could be attributed to the degradation of the amorphous zones in PLA. This would be in good agreement with the severe disintegration observed in Figure 10, and the large reduction in the intrinsic viscosity shown in Table 4. Furthermore, an additional melting event at a lower temperature than the melting point of PLA before composting was observed as a shoulder, which could be ascribed to the presence of shorter polymer chains due to the degradation of the PLA matrix [69].

The chemical and structural changes in the materials before and after 11 days of composting were also followed by ATR-FTIR. The spectra of analyzed materials after 11 days under composting conditions (Figure 12) showed important differences with respect to those of the starting materials. On the one hand, in the spectrum region between 1750 and 1500 cm^−1^, all the materials subjected to composting conditions for 11 days showed a shoulder around 1700 cm^−1^, assigned to the formation of -COOH groups during hydrolytic degradation [70]. Moreover, a small but broad band centered at 1600 cm^−1^ appeared in PLAR, which is related to the presence of carboxylate ions in degraded PLA formulations [69,71]. It is also worth noting that, in the PLAR–SFNs nanocomposite, the analysis of the Amide I and II regions of the ATR-FTIR spectra of silk fibroin materials can be used as the most informative for their secondary structure determination [35,72,73]. Figure 12d shows that, although at day 0 both bands were hindered by the PLAR spectrum due their low SFN/PLAR ratio, a remarkable increase in the signals of these SFNs bands is appreciated after 11 days. The spectra of silk fibroin nanoparticles, mainly in a β-sheet, highly crystalline conformation (amide I at ~1626 cm^−1^ and amide II at ~1520 cm^−1^), and the regenerated silk fibroin (non-crystalline or Silk I), which was mainly in a random coil (RC) conformation (amide I at ~1650 cm^−1^ and amide II at ~1538 cm^−1^), are presented in the figure as a reference. In both cases, the signals are composed by the sum of the contributions of the random coil and β-sheet conformations. This behavior could be explained by the fact that the decomposition of the PLAR makes accessible the crystallized silk fibroin (highly ordered, insoluble, and stable) present in the nanoparticles to the ATR, arising from the background of the PLAR spectrum. However, the SFNs have been partially decomposed during this period into soluble peptides, mainly in a “random coil” conformation, which are visible too [74]. Finally, it should be noted that the spectrum of the PLAR–YMNs nanocomposite was the most similar to that of virgin material in good agreement with the lower disintegration rate of this formulation at this stage due to the protective effect of YMNs.

On the other hand, all composted materials after 11 days showed an increase in the intensity of the absorption bands at 1211 and 920 cm^−1^, along with a decrease in the intensity of the absorption band at 1265 cm^−1^ and 956 cm^−1^. The bands at 1211 cm^−1^ (ν_as_(C-CO-O) + r_as_(CH_3_)) and 920 cm^−1^ (assigned above) have been associated in the literature with the formation of crystalline polymorphs, while the bands at 1265 cm^−1^ ((ν(C-H)+ ν(C-CO-O)) and at 956 cm^−1^ correspond to the amorphous phase [53]. The observed results suggest that the amorphous phase considerably decreased due to the earlier disintegration of the amorphous regions, while the crystalline regions remained in the polymer matrix as was already pointed out for DSC results [71].

After 17 days under composting conditions, tiny pieces of polymeric samples were recovered (Figure 10) and all the formulations had lost more than 90% of their initial mass, which is frequently used as the goal of the disintegrability test [68,71]. After 23 days, the materials had completely disappeared, and the compost soil showed a pH around 7. Moreover, the compost soil’s color changed during the composting test, as is shown at the bottom of Figure 10, resulting in a dark humus soil as a consequence of the aerobic fermentation, confirming the success of the composting test.

## 4. Conclusions

Nanocomposites based on recycled PLA reinforced with 1 wt.% of silk fibroin or yerba mate nanoparticles were developed and exposed to two ethanolic food simulants containing 10% *v*/*v* of ethanol (simulant A) and 50% *v*/*v* of ethanol (simulant D1). The migration levels of the nanocomposites were below the overall migration limits established in the current legislation, suggesting that these recycled films can be used as food packaging materials. The effects of ethanol on the structural, thermal, and mechanical properties of the developed materials after the overall migration tests were studied. After the immersion in food simulants, the materials showed an increase in the Vickers hardness that was more marked in the simulant D1, directly related to the larger increase in the crystallinity degree in this food simulant as was observed by DSC and ATR-FTIR spectroscopy. Finally, the materials were subjected to simulated composting conditions, and they were successfully disintegrated in less than one month, underlining their biodegradable character. The ability of both nanoparticles to act as reinforcing fillers for recycled PLA slowed down its disintegration rate under composting conditions due to the smaller reduction in the molecular weight of the polymeric matrix, as revealed by the intrinsic viscosity and ATR-FTIR measurements.

The obtained results suggest that recycled PLA performance can be improved by the addition of 1 wt.% of either SFNs or YMNs, while the obtained nanocomposites are promising materials for sustainable food packaging applications.

## Figures and Tables

**Table 1:** Samples obtained after the mechanical recycling process.

Sample	Description
PLAV	PLA films obtained from extrusion and compression molding of PLA pellets.
PLAR	PLAV films subjected to accelerated aging, washing, and reprocessing.
PLAR-SFN	PLAR with 1 wt.% of silk fibroin nanoparticles.
PLAR-YMN	PLAR with 1 wt.% of Yerba mate nanoparticles.

**Table 2:** DSC first heating results and FTIR crystallinity degree of the different samples.

Sample	*T_g_* (°C)	*T_cc_* (°C)	*T_m_* (°C)	∆*H_cc_* (J/g)	∆*H_m_* (J/g)	$X_{C}^{DSC}$ (%)	$X_{C}^{FTIR}$ (%)
**No immersion**							
PLAV	57.7	116.8	149.9	25.8	25.9	0	0
PLAR	56.9	106.4	147.5–155.2	25.1	25.9	1	0
PLAR–SFNs	55.6	106.6	146.6–153.4	24.8	27.5	3	0
PLAR–YMNs	55.2	105.8	146.2–153.4	25.2	26.4	1	0
**After immersion in food simulant A**							
PLAV	57.4	101.6	145.6–153.3	24.0	28.4	5	0
PLAR	56.4	91.4	143.9–152.1	23.0	28.5	6	0
PLAR–SFNs	58.9	98.7	145.2–153.1	22.5	27.3	5	7
PLAR–YMNs	60.7	97.8	145.1–152.3	21.3	26.0	5	0
**After immersion in food simulant D1**							
PLAV	53.5	Not shown	141.4–153.0	0	34.4	37	34
PLAR	53.1	Not shown	141.4–153.0	0	32.2	35	33
PLAR–SFNs	53.6	Not shown	153.1	0	32.6	35	31
PLAR–YMNs	54.6	Not shown	152.8	0	32.3	35	24

**Table 3:** TGA parameters of the samples before and after immersion in food simulants.

Sample	T_10_ (°C)	T_50_ (°C)	T_max_ (°C)
**No immersion**			
PLAV	328.2	348.9	354.5
PLAR	323.1	350.2	359.5
PLAR–SFNs	331.2	360.4	370.0
PLAR–YMNs	326.8	352.6	359.5
**After immersion in simulant A**			
PLAV	327.3	343.5	347.4
PLAR	323.6	350.8	356.0
PLAR–SFNs	325.9	353.4	362.8
PLAR–YMNs	318.7	348.5	357.8
**After immersion in simulant D1**			
PLAV	324.2	341.4	345.5
PLAR	313.4	341.0	348.5
PLAR–SFNs	327.5	353.2	359.6
PLAR–YMNs	319.3	349.0	356.6

**Table 4:** Intrinsic viscosity and TGA parameters of the samples during disintegration under composting conditions.

Sample	Disintegration Time (Day)	Intrinsic Viscosity (mL/g)	T_10_ (°C)	T_max_ (°C)
PLAV	0	159 ± 1	328.2	354.5
	4	93 ± 2	282.5	319.3
	11	24 ± 1	266.1	339.2
PLAR	0	135 ± 1	323.1	359.5
	4	77 ± 1	295.5	333.6
	11	17 ± 1	263.1	340.5
PLAR–SFNs	0	119 ± 2	331.2	369.1
	4	79 ± 3	312.0	344.1
	11	27 ± 1	301.7	336.9
PLAR–YMNs	0	133 ± 5	317.9	355.2
	4	90 ± 3	302.8	335.3
	11	32 ± 1	296.0	336.9

## Data Availability

Research data are available from the public repository e-cienciadatos https://doi.org/10.21950/QOTKHI (accessed on 7 June 2021).

## Supplementary Materials

The following are available online at https://www.mdpi.com/article/10.3390/polym13121925/s1, Figure S1: (**a**) DLS measurements of YMNs solution and powder and (**b**) TEM image of YMNs powder; Figure S2: (**a**) DLS measurements of SFNs and (**b**) TEM image of SFNs powder and Figure S3: FE-SEM images of: PLAV (**a**), PLAR (**b**), and nanocomposites: PLAR loaded with 1% wt. of YMN (**c**) and PLAR loaded with 2% wt. of SFN (**d**).
