# Supplementary material for: Effect of Yerba Mate and Silk Fibroin Nanoparticles on the Migration Properties in Ethanolic Food Simulants and Composting Disintegrability of Recycled PLA Nanocomposites
Source: Polymers (Basel). 2021 Jun 10;13(12):1925. doi: 10.3390/polym13121925 (PMC8230047; doi:10.3390/polym13121925)
Supplement: Supplementary file 1 [file polymers-13-01925-s001.zip › polymers-1242994-supplementary.pdf]

polymers

MDPI

Supporting Information

Effect of Yerba Mate and Silk Fibroin Nanoparticles on the Migration Properties in Ethanolic Food Simulants and Composting Disintegrability of Recycled PLA Nanocomposites

Freddys R. Beltrán [1,2], Marina P. Arrieta [1,2,*], Diego Elena Antón [1], Antonio A. Lozano-Pérez [3], José L. Cenis [3], Gerald Gaspar [1,2], María U. de la Orden [2,4] and Joaquín Martínez Urreaga [1,2]

[1] Departamento de Ingeniería Química Industrial y Medio Ambiente, Universidad Politécnica de Madrid, E.T.S.I. Industriales, 28006 Madrid, Spain; f.beltran@upm.es (F.R.B.); d.elena@alumnos.upm.es (D.E.A.); geraldmanuel.gaspar@upm.es (G.G.); joaquin.martinez@upm.es (J.M.U.)

[2] Grupo de Investigación Polímeros Caracterización y Aplicaciones (POLCA), Madrid, Spain; mariula@ucm.es

[3] Depertamento de Biotecnología, Genómica y Mejora Vegetal, Instituto Murciano de Investigación y Desarrollo Agrario y Alimentario (IMIDA), 30150 Murcia, Spain; abel@um.es, abel@um.es (A.A.L.-P.); josel.cenis@carm.es (J.L.C.)

[4] Deparamento de Química Orgánica, Facultad de Óptica y Optometría, Universidad Complutense de Madrid, 28037 Madrid, Spain

* Correspondence: m.arrieta@upm.es; Tel.: +34-910-677-301

Instrumental

The hydrodynamic size of either SFN or YMNs was measured by means of dynamic light scattering (DLS). The obtained particles, in the powder form, were dispersed at 1 mg·mL^{-1} in ultrapure deionized water by ultrasonication at 10% of amplitude for 1 minute with a Branson SFX550 (Emmerson Ultrasonic Corporation. Dansbury, USA) and further measured at 20 °C in a Zetasizer Nano series ZSP equipment (Malvern Instrument Ltd., Malvern, UK).

Transmission electron microscopy (TEM) measurements of SFN and YMNs were carried out on a Zeiss EM902 (Zeiss, Oberkochen, Germany) and a JEOL JEM-1010 (JEOL Ltd., Tokyo, Japan) operating at 100kV, respectively. YMNs suspension was prepared by dissolving 1 mg of YMNs powder into 1 mL of water. One droplet of YMNs suspension (1 mg mL^{-1}) was deposited on carbon-coated copper grids and dried at room temperature during 25 min before TEM observation.

The cryo-fractured surface microstructure of the cross section of films was observed by means of field emission scanning electron microscopy (FE-SEM). Films were previously sputtered with a palladium/gold layer. PLAV, PLAR and PLA-YMN were observed in a JEOL JSM 7600F microscope (JEOL Ltd., Tokyo, Japan) operating at 5 kV and nanocomposite of PLAR loaded with 2% SFN was observed in a FE-SEM S8000 (Hitachi, Tokyo, Japan) at 20kV

Results

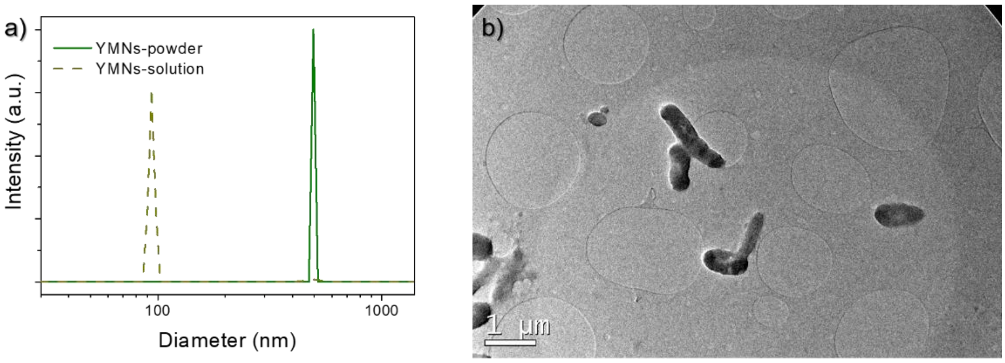

Figure S1. (**a**) DLS measurements of YMNs solution and powder and (**b**) TEM image of YMNs powder.

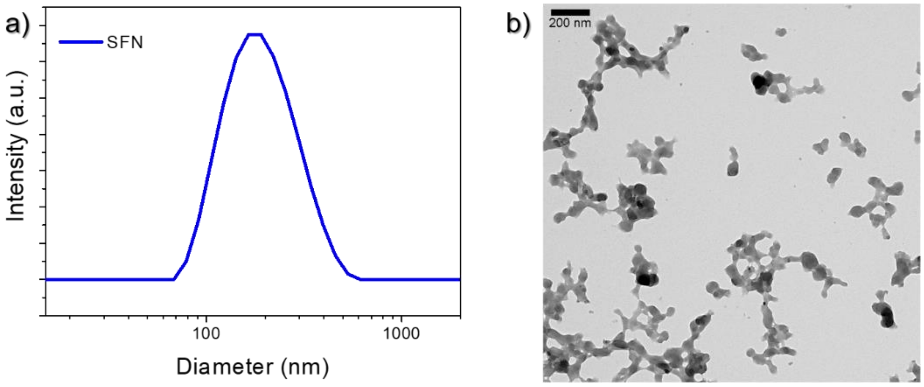

Figure S2. (**a**) DLS measurements of SFNs and (**b**) TEM image of SFNs powder.

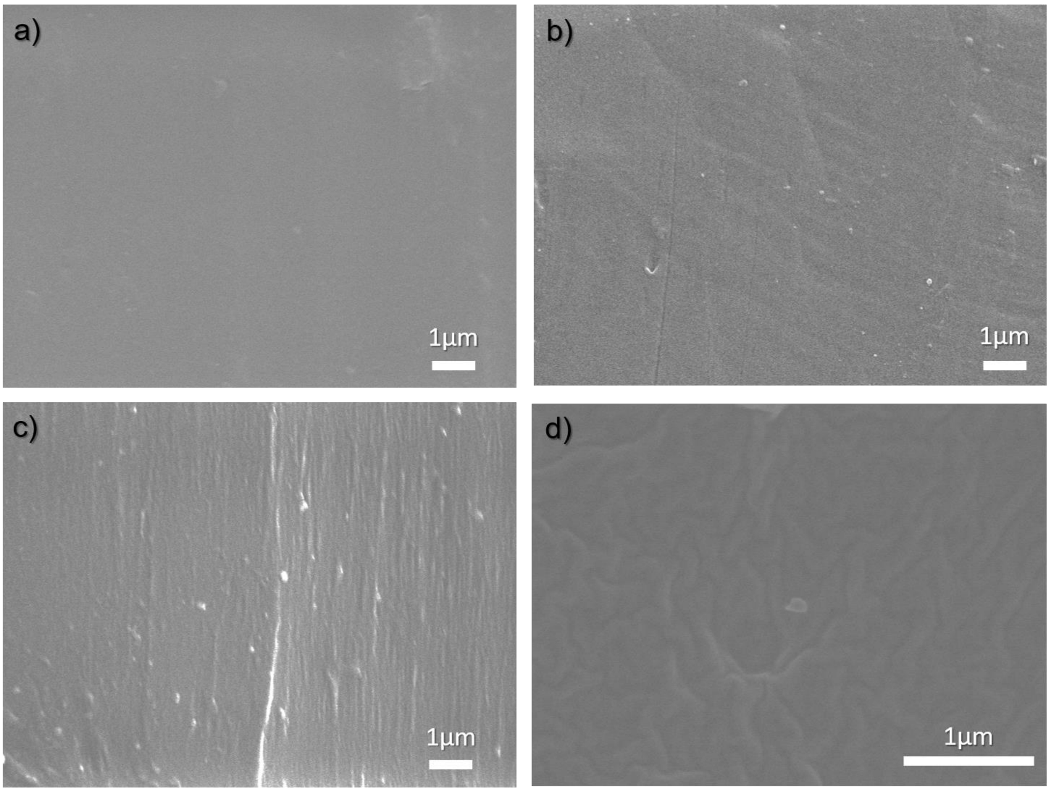

Figure S3. FE-SEM images of: PLAV (**a**), PLAR (**b**), and nanocomposites: PLAR loaded with 1% wt. of YMN (**c**) and PLAR loaded with 2% wt. of SFN (**d**).
